# Loss of CAMKK2 and iron-transport proteins—transferrin and its receptor—in the Alzheimer’s disease hippocampus: link to tau pathology

**DOI:** 10.3389/fcell.2026.1716718

**Published:** 2026-01-22

**Authors:** Mohammad Golam Sabbir, Behzad Mansouri, Bram Ramjiawan

**Affiliations:** 1 Department of Psychology and Neuroscience, College of Psychology, Nova Southeastern University, Davie, FL, United States; 2 Alzo Biosciences Inc., San Diego, CA, United States; 3 Neuroptek and Brain, Vision, and Concussion Clinic, Winnipeg, MB, Canada; 4 Department of Pharmacology and Therapeutics, Max Rady College of Medicine, Faculty of Health Sciences, University of Manitoba, Winnipeg, MB, Canada

**Keywords:** Alzheimer’s disease, Ca^2+^/calmodulin (CaM)-dependent protein kinase kinase 2, calcium, homeostasis, iron, Parkinson’s disease, transferrin, transferrin receptor

## Abstract

**Introduction:**

Calcium and iron are essential bioelements regulating neuronal function and survival. Dysregulation of calcium signaling and iron homeostasis is implicated in Alzheimer’s disease (AD), contributing to oxidative stress, synaptic dysfunction, and neurodegeneration. Previously, using *in vitro* cell-based models and transgenic mice, we demonstrated that CAMKK2, a calcium/calmodulin-dependent protein kinase, regulates iron transport via transferrin (TF) and transferrin receptor (TFRC). While excessive iron deposition is a hallmark of AD brains, the mechanisms underlying its dysregulation remain poorly understood. In a prior study of postmortem temporal cortex tissues, we showed that CAMKK2/TF/TFRC protein levels were significantly reduced in AD compared to cognitively normal (CN) individuals, and that increased iron accumulation in AD correlated with reduced TF/TFRC levels. This follow-up study aimed to assess CAMKK2/TF/TFRC protein levels in hippocampal tissues - an early site of AD pathology - and examine their relationship with tau (MAPT) aggregation in AD, Parkinson’s disease (PD), and frontotemporal dementia (FTD).

**Methods:**

Postmortem hippocampal tissues from 29 CN individuals and patients diagnosed with AD/FTD/PD (N = 73/7/9 respectively) were analyzed. CAMKK2/TF/TFRC/MAPT levels were quantified using Western blotting. Correlation analyses evaluated associations among these proteins and with age, sex, and postmortem interval (PMI). Isoelectric focusing (IEF) was used to assess post-translational modifications of CAMKK2 and TF.

**Results:**

CAMKK2 and TF levels were significantly reduced in AD, FTD, and PD hippocampi compared to CN controls. TFRC reduction was specific to late onset AD, suggesting a later event. MAPT levels were significantly elevated in AD, with high molecular weight smears indicating tau aggregation. CAMKK2 and MAPT were positively correlated in CN but not in AD, indicating disease-specific disruption. TF and CAMKK2 were also positively correlated in CN but attenuated in AD. No significant changes in CAMKK2 or TF charge states were detected.

**Discussion:**

CAMKK2 downregulation and impaired iron transport appear to be shared features across multiple neurodegenerative diseases, but their decoupling from tau pathology seems specific to AD. These findings position CAMKK2 as a molecular gatekeeper linking calcium signaling, iron metabolism, and tau aggregation. Future studies should focus on elucidating the mechanisms underlying CAMKK2 downregulation to better understand its role in AD pathogenesis.

## 1 Introduction

Iron (Fe) and calcium (Ca) are essential bioelements that play critical roles in maintaining neuronal function and cellular homeostasis in the brain. Iron contributes to a wide range of fundamental biological processes, including oxygen transport, DNA synthesis, mitochondrial respiration, myelin formation, and neurotransmitter synthesis and metabolism (Crichton and Boelaert, 2001). Calcium, primarily in its ionic form (Ca^2+^), functions as a universal second messenger, regulating diverse signaling pathways and cellular activities such as neurotransmitter release (Südhof, 2012), gene expression (Puri, 2020), synaptic plasticity (Lamont and Weber, 2012), cell death (Orrenius et al., 2003), and neurodegeneration (Mattson, 2007). Emerging evidence suggests that calcium signaling and iron homeostasis are intricately linked, particularly in the context of neuronal health and disease. For instance, iron-induced oxidative stress can impair the function of Ca^2+^-regulating proteins, leading to disruptions in calcium signaling and triggering apoptotic cell death (Núñez et al., 2019; Hidalgo and Donoso, 2008; Görlach et al., 2015). Conversely, disturbances in Ca^2+^ homeostasis may affect Ca^2+^-dependent proteins located on cellular and organelle membranes, facilitating the influx of Fe^2+^ or release from intracellular stores contributing to iron overload and ferroptotic neuronal death (Gleitze et al., 2021; Xu et al., 2024). These bidirectional interactions underscore the importance of maintaining balanced Ca^2+^ signaling and iron homeostasis for neuronal survival.

In neurodegenerative diseases such as Alzheimer’s disease (AD), dysregulation of both iron (Mohammadi et al., 2024; Gleason and Bush, 2021; Ayton et al., 2020; van Duijn et al., 2017; Smith et al., 1997) and Ca^2+^ (Berridge, 2010; Marambaud et al., 2009; Baracaldo-Santamaría et al., 2023; Webber et al., 2023) has been implicated in disease progression. Iron accumulation in the brain promotes oxidative damage and neurotoxicity, while Ca^2+^ signaling abnormalities compromise synaptic integrity and neuronal viability. Iron is primarily transported by the serum protein transferrin (TF), which delivers iron to cells via transferrin receptor (TFRC)-mediated endocytosis. Although alternative iron transport mechanisms exist, receptor-mediated uptake of TF-bound iron is considered the major pathway by which the brain acquires iron from peripheral circulation across the blood–brain barrier (BBB) (Gkouvatsos et al., 2012; Knutson, 2017).

In our previous studies using CRISPR/Cas9-mediated CAMKK2 knockout human cell lines and Camkk2-deficient mouse models, we demonstrated that Ca^2+^/calmodulin (CaM)-dependent protein kinase 2 (CAMKK2), a key mediator of Ca^2+^ signaling, regulates TF and TFRC-mediated iron transport and homeostasis (Sabbir et al., 2020; Sabbir, 2020; Sabbir, 2018). Furthermore, we showed that CAMKK2 mediates TF trafficking through its downstream kinase CAMK4 (Sabbir, 2020). Supporting this, a large-scale cross-study transcriptomic analysis of late-onset AD brains (129 samples) and age-matched non-demented controls (101 samples) revealed a significant reduction in CAMK4 expression in the prefrontal cortex of AD patients (Mean ± SEM: 0.3276 ± 0.2751 vs. 0.1187 ± 0.1458; *p* < 0.0001; GEO accession number: GSE44770) (Zhang et al., 2013). Additionally, our postmortem human tissue-based study showed that CAMKK2 protein levels are significantly reduced in the temporal cortex of AD patients (n = 74) compared to age-matched cognitively normal (CN) individuals (n = 17) (Sabbir, 2024). This reduction was associated with decreased TF and TFRC levels and increased iron content, suggesting a mechanistic link between Ca^2+^ signaling disruption, CAMKK2/CAMK4 loss-of-function, and iron dyshomeostasis in AD (Sabbir, 2024).

Despite the known vulnerability of the hippocampus in early AD pathology (Halliday, 2017; Mu and Gage, 2011), the role of CAMKK2-TF/TFRC signaling in this region remains poorly understood. The hippocampus is central to learning and memory and is among the first regions to exhibit tau pathology and neuronal loss in AD. Recent single-nucleus RNA sequencing studies of the human hippocampus have demonstrated overlapping expression of CAMKK2, TF, and TFRC across multiple hippocampal cell subtypes, including neurons, astrocytes, oligodendrocytes, and microglia (Su et al., 2022; Speir et al., 2021). Therefore, to determine whether CAMKK2 dysregulation and iron transport protein loss are also observed in the hippocampus during AD pathogenesis - as was previously reported in our temporal cortex tissue-based study (Sabbir, 2024) - the abundance and interrelationships of CAMKK2, TF, TFRC, and microtubule-associated protein tau (MAPT) were investigated in postmortem hippocampal tissues obtained from cognitively normal individuals (CN; N = 29) and patients diagnosed with early-onset AD (EOAD; N = 42), late-onset AD (LOAD; N = 31), frontotemporal dementia (FTD: N = 7), and Parkinson’s disease (PD; N = 9). FTD and PD were included as neurodegenerative disease controls to help distinguish AD-specific molecular changes from those that may be shared across multiple neurodegenerative conditions. These molecular signatures were examined, and correlations with MAPT aggregation, age, and sex were assessed to provide deeper insight into the molecular mechanisms underlying hippocampal vulnerability in AD.

## 2 Materials and methods

### 2.1 Human brain tissue collection

Frozen hippocampal tissue samples from CN individuals and patients diagnosed with AD, FTD, and PD were obtained from the NIH NeuroBioBank repositories (Request ID #1883). The cohort included CN individuals (N = 29), EOAD (N = 42), LOAD (N = 31), FTD (N = 7), and PD (N = 9). Classification of AD cases into EOAD and LOAD was provided by the NIH NeuroBioBank and is publicly accessible at https://neurobiobank.nih.gov. Additional hippocampal samples representing various developmental and aging stages - from prenatal to elderly - were generously provided by Dr. Richard Deth through the NIH NeuroBioBank. This developmental cohort included 9 CN individuals spanning a wide age range (Supplementary Table 1). All tissue samples were stored at −80 °C until further analysis. Detailed metadata for these samples are available in previously published supplementary tables (Sabbir et al., 2022).

The NIH NeuroBioBank provides essential demographic and diagnostic information (e.g., age, sex, diagnosis, postmortem interval) for each sample; however, detailed clinical metadata such as patient life history, pharmacological treatment, or disease stage-specific interventions are not available. Therefore, treatment history could not be considered in sample selection or analysis, and future studies using cohorts with comprehensive clinical records are needed to evaluate the potential impact of therapeutic interventions on CAMKK2 and iron transport pathways.

### 2.2 Protein analysis via western blotting

Hippocampal tissue samples were homogenized in ice-cold lysis buffer (50 mM Tris-HCl, pH 7.4; 150 mM NaCl; 1% NP-40; 0.5% sodium deoxycholate) supplemented with protease and phosphatase inhibitors (Thermo Fisher Scientific). Lysates were centrifuged at 16,000 × g for 15 min at 4 °C, and supernatants were collected for protein quantification. For each lane, 30 µg of total protein was loaded onto 10%–14% SDS-PAGE gels. Electrophoresis was performed at 120–180 V until the dye front reached the bottom of the gel. Proteins were transferred to 0.2 µm nitrocellulose membranes using a Trans-Blot® SD Semi-Dry Transfer Cell system (Bio-Rad) at 25 V for 15 min using transfer buffer provided with the kit. Membranes were blocked in 5% non-fat dry milk in TBST (Tris-buffered saline with 0.5% Tween-20) for 1 h at room temperature, followed by overnight incubation at 4 °C with primary antibodies diluted in blocking buffer: CAMKK2 (1:500; Santa Cruz Biotechnology, sc-100364), TF (1:1000; Santa Cruz Biotechnology, sc-365871), TFRC (1:1000; Santa Cruz Biotechnology, sc-51829), MAPT (1:1000; Santa Cruz Biotechnology, sc-58860), and GAPDH (1:5000; Santa Cruz Biotechnology, sc-25778). The CAMKK2, TF, TFRC, MAPT, and GAPDH antibodies have been validated in previous studies (Sabbir et al., 2020; Sabbir et al., 2022; Sabbir et al., 2023a; Sabbir et al., 2023b). After washing, membranes were incubated with HRP-conjugated secondary antibodies (1:5000) for 1 h at room temperature. Immunoreactive bands were visualized using enhanced chemiluminescence (ECL; Bio-Rad Clarity™ Western ECL Substrate) and imaged with a Bio-Rad ChemiDoc™ MP Imaging System under identical exposure settings for all samples within an experiment.

Band intensities were quantified using ImageJ software. To ensure consistency across experiments, blots probed with anti-CAMKK2, anti-TF, and anti-TFRC antibodies were normalized to a reference lysate derived from wild-type HepG2 cells, which endogenously express all three proteins. A large batch of HepG2 cells was lysed and aliquoted to serve as standardized internal control across all blots. Additionally, CAMKK2 knockout HepG2 cells were used as a negative control for CAMKK2 detection and as a positive control for TF and TFRC, as their expression levels are known to increase upon CAMKK2 deletion (Sabbir, 2018). This internal standardization strategy, previously employed in several of our studies, allowed for reliable quantification across multiple blots performed under varying experimental conditions. To ensure reproducibility and consistency of immunoblot data, each sample was run in triplicate or quadruplicate within the same gel. A large-format slab gel with 26 lanes was used to accommodate multiple replicates and sample types simultaneously. After electrophoresis, the gel was carefully sectioned, and two gel segments containing different samples were transferred onto a single large nitrocellulose membrane. This strategy allowed for high throughput immunoblotting with maximal sample representation and internal consistency. The use of multiple replicates and standardized membrane loading has been consistently applied throughout our studies to enhance the reliability and reproducibility of protein quantification.

Due to the absence of MAPT expression in HepG2 cells, a different quantification approach was used for MAPT. Blots for MAPT and GAPDH were generated using the same protein loading and imaged simultaneously under identical exposure settings. MAPT signal intensities were then normalized to GAPDH to account for loading differences. This ratio-based method enabled consistent quantification of MAPT across multiple blots and experimental runs. All antibodies used in this study have been validated in prior publications.

### 2.3 Isoelectric focusing (IEF)

Isoelectric focusing was performed following a protocol adapted from previously established methods (Sabbir, 2018; Sabbir et al., 2022; Sabbir et al., 2023a). In brief, 50 μg of total protein lysate from hippocampus tissue samples was precipitated using acetone and subsequently resuspended in a rehydration buffer containing 8 M urea, 2% CHAPS, 50 mM dithiothreitol (DTT), and 0.2% Bio-Lyte ampholytes (pH 3–10). The protein solution was loaded onto immobilized pH gradient (IPG) strips (non-linear, Bio-Rad) and allowed to rehydrate overnight. Focusing was performed in three steps: an initial hold at 175 V for 15 min, a voltage ramp from 175 V to 2,000 V over 45 min, followed by a final focusing stage at 2,000 V for 30 min. After focusing, the IPG strips were sequentially treated with DTT for reduction and iodoacetamide for alkylation. Proteins were then resolved in the second dimension by SDS-PAGE and analyzed by immunoblotting.

### 2.4 Statistical analysis

All statistical analyses were conducted using GraphPad Prism (version 10.6.0). Protein expression data were normalized to a designated reference sample as explained before. Group comparisons were performed using unpaired t-tests or one-way ANOVA with Dunnett’s *post hoc* test. Correlation analyses employed Pearson’s correlation or linear regression, assuming normal distribution of variables. Statistical significance was defined as *p* < 0.05.

## 3 Results

### 3.1 CAMKK2 TF, and TFRC are expressed in different hippocampal cell types

**FIGURE 1:**
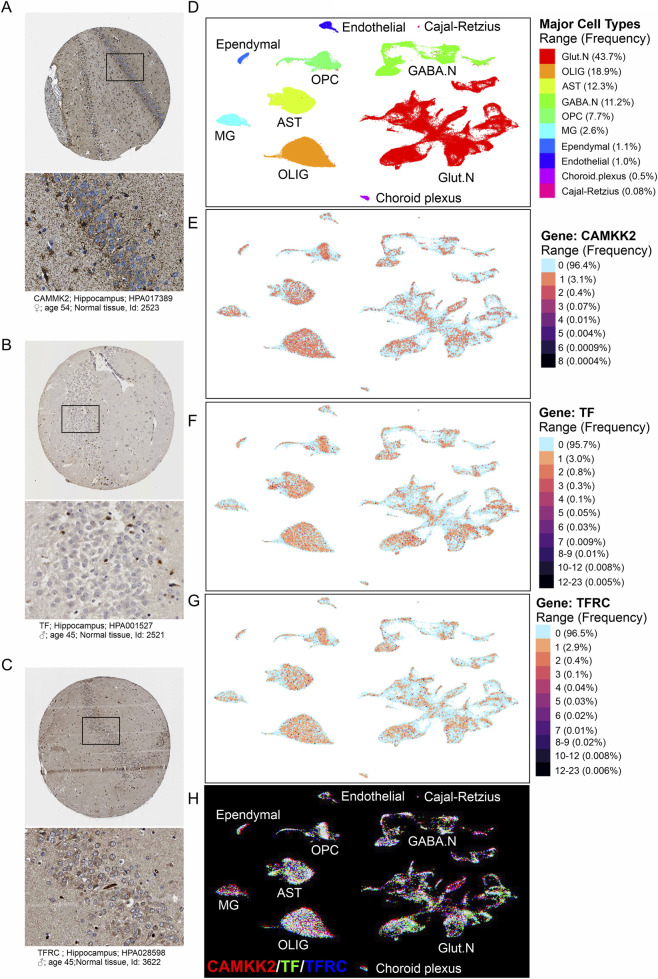
Expression of CAMKK2, TF, and TFRC in the human hippocampus. **(A–C)** Immunohistochemistry images showing CAMKK2, TF, and TFRC protein expression in hippocampal neurons. Data were obtained from archived images in the Human Protein Atlas accessed on 19th September 2025. **(D)** UCSC Cell Browser interface displaying hippocampal cell clusters identified by single-nucleus RNA (snRNA) sequencing of the postnatal human hippocampus across ages (Su et al., 2022). Ten major cell types were annotated by Su et al. using known marker genes, including glutamatergic neurons (Glut.N), oligodendrocytes (OLIG), astrocytes (AST), oligodendrocyte progenitor cells (OPCs), GABAergic neurons (GABA.N), and microglia (MG), along with less abundant (<1%) populations such as endothelial cells, ependymal cells, choroid plexus cells, and Cajal-Retzius cells. The total number of nuclei analyzed was 224,464 (Su et al., 2022). **(E–G)** UCSC Cell Browser views showing the same cell clusters, colored by expression levels of CAMKK2 **(E)**, TF **(F)**, and TFRC **(G)**. Higher expression levels are indicated in dark red. A legend on the right side of the interface maps color intensity to expression bins and shows the frequency of cell types with similar expression levels. **(H)** Using the dataset shown in panels **(E–G)**, the cell-type-specific expression profiles of CAMKK2, TF, and TFRC are false-colored and superimposed to illustrate their overlapping expression patterns across diverse hippocampal cell types.

The expression patterns of CAMKK2, transferrin (TF), and transferrin receptor (TFRC) in the adult human hippocampus have been documented using data from the Human Protein Atlas (HPA) (Uhlén et al., 2015) and the UCSC Cell Browser (Speir et al., 2021). Immunohistochemistry (IHC) data from the HPA revealed the presence of CAMKK2 (Figure 1A), TF (Figure 1B), and TFRC (Figure 1C) in both neuronal and glial populations within postmortem hippocampal tissue. Complementary single-nucleus RNA sequencing data from the UCSC Cell Browser, specifically the *Human Hippocampus Across the Postnatal Lifespan* dataset (Su et al., 2022; Speir et al., 2021), demonstrated overlapping expression of CAMKK2, TF, and TFRC across multiple hippocampal cell subtypes (Figures 1D–H). These include glutamatergic neurons (Glut.Ns), GABAergic neurons (GABA.Ns), astrocytes (ASTs), oligodendrocytes (OLIGs), oligodendrocyte progenitor cells (OPCs), and microglia (MG). Additionally, low-level expression (<1%) was observed in endothelial cells, ependymal cells, and choroid plexus cells (Figures 1D–H).

Collectively, these findings suggest that CAMKK2, TF, and TFRC are broadly expressed across nearly all major hippocampal cell types, supporting their potential involvement in diverse cellular processes relevant to hippocampal function and AD pathology.

### 3.2 Characterization of AD, PD, FTD hippocampus tissue cohorts using microtubule-associated protein tau (MAPT) profiling

**FIGURE 2:**
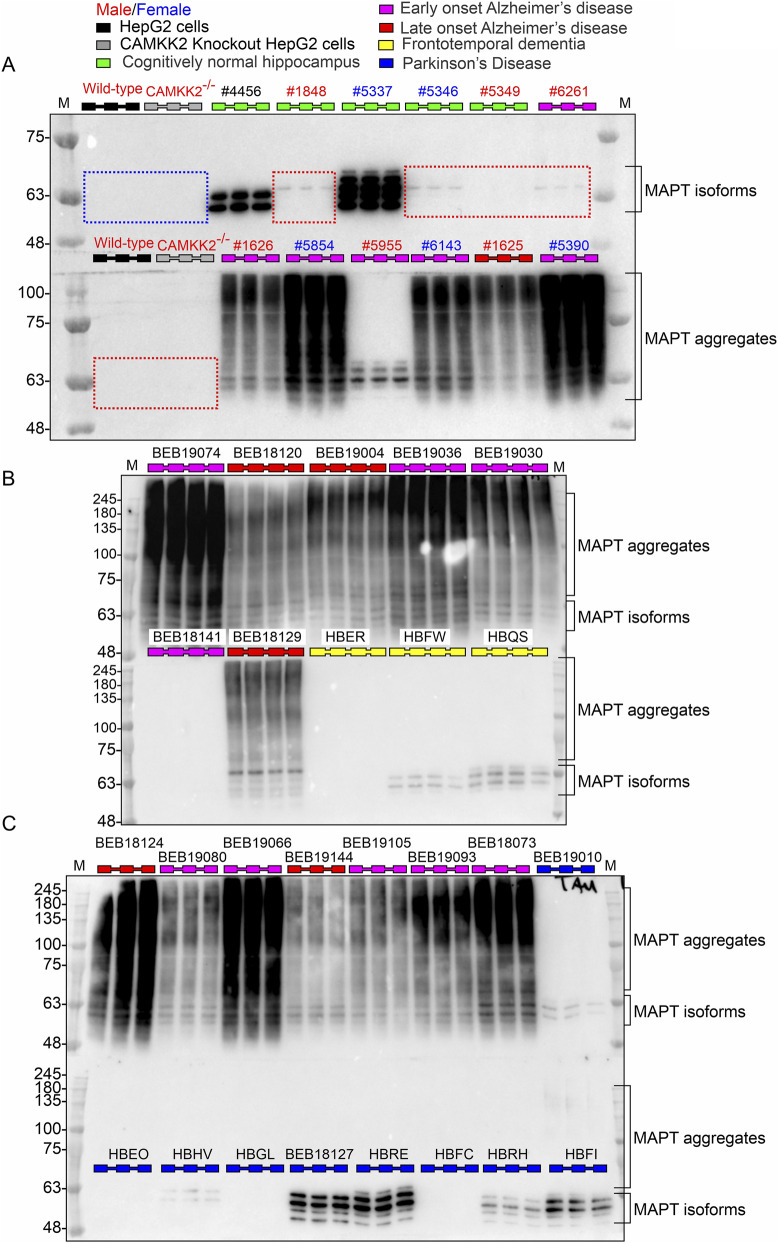
Abundance of MAPT in hippocampal tissues from CN, EOAD, LOAD, FTD, and PD individuals (postmortem). **(A–C)** Representative immunoblots showing MAPT protein levels in postmortem hippocampal tissues from CN, EOAD, LOAD, FTD, and PD individuals. Wild-type HepG2 cells and CAMKK2-knockout HepG2 cells were used as positive and negative controls, respectively, for CAMKK2, TF, and TFRC proteins, depending on the context, and are referenced in subsequent figures. Blue rectangles indicate the absence of MAPT expression in HepG2 cells. Red dotted rectangles highlight low expression of MAPT isoforms in select hippocampal samples from both non-demented and neurodegenerative cases. Notably, multiple MAPT isoforms are expressed in most CN, FTD, and PD tissues, whereas in EOAD and LOAD samples, MAPT appears predominantly as a smear, suggesting post-translational modifications and formation of protein aggregates. Each sample was loaded in triplicates or quadruplicates. Note: GAPDH immunoblots for the samples shown here are presented in Figure 8. The same hippocampal samples were also used in subsequent representative immunoblots for CAMKK2, TF, and TFRC. Due to space constraints, representative GAPDH blots were omitted from this figure, as they are already provided to support consistent loading and quantification.

**FIGURE 3:**
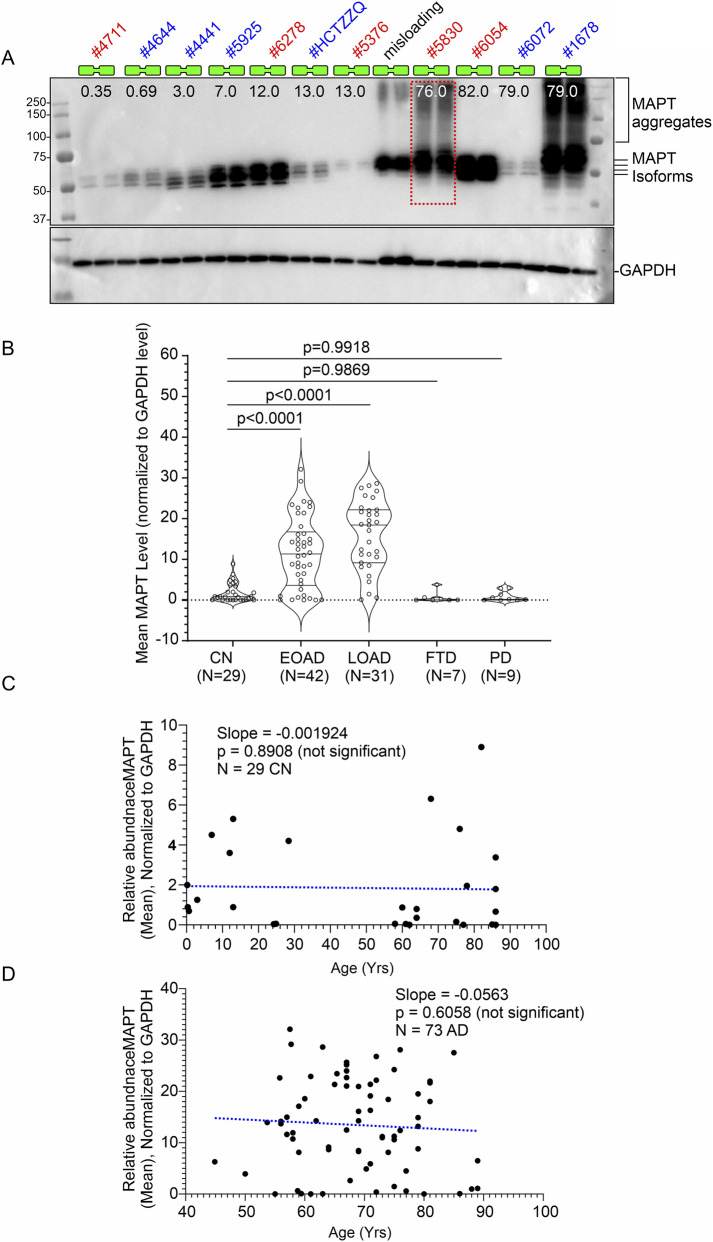
Age-related abundance of MAPT in hippocampal tissues from CN individuals and quantitative comparison with AD patients (postmortem). **(A)** Representative immunoblots showing MAPT and corresponding GAPDH protein levels in postmortem hippocampal tissues from CN individuals across different age groups. The age at death for each individual is indicated (in years) in the top panel below the sample numbers. Each sample was loaded in duplicates. **(B)** Scatter plots showing MAPT protein levels normalized to GAPDH in hippocampal tissues from 29 CN individuals, 42 EOAD, 31 LOAD, 7 FTD, and 9 PD patients (postmortem). The mean MAPT abundance was calculated by dividing the intensity of the MAPT band by the intensity of the GAPDH band in the same lane, using consistent exposure settings across all experiments. The final values represent the average from three independent immunoblotting experiments, each with 3–4 replicates using the same tissue lysates. Data are presented as Mean ± SEM. Statistical significance was assessed using Ordinary One-Way ANOVA followed by Dunnett’s Multiple Comparisons test. **(C)** MAPT abundance plotted against age in CN individuals. **(D)** MAPT abundance plotted against age in AD patients. Regression lines were generated using GraphPad Prism software.

Hippocampal tissues from a cohort of 29 CN individuals, 42 EOAD cases, 31 LOAD cases, 7 FTD cases, and 9 PD cases were utilized to examine the protein abundance of CAMKK2, TF, and TFRC. Diagnostic classification of these samples was performed by NIH Neurobiobank. To further verify the presence of Alzheimer’s pathology, MAPT immunoblotting was performed, based on the premise that neurofibrillary tangles (NFTs) - a hallmark of AD - are composed of aggregated tau protein and are expected to be present in AD tissues compared to others. These aggregates typically appear as high molecular weight smears on Western blots, reflecting polymerization of the six MAPT isoforms (45–65 kDa) (Sabbir, 2024). The immunoblots revealed multiple MAPT bands in the 45–70 kDa range across CN (Figure 2A), FTD (Figure 2B), and PD (Figure 2C) samples, consistent with baseline tau expression. In several samples - including those from CN, AD, FTD, and PD groups - MAPT immunoreactivity was either faint or undetectable, even under high exposure conditions (Figures 2A–C, red dotted squares), suggesting low expression levels or absence within detection limits. In contrast, most EOAD and LOAD samples exhibited distinct high molecular weight MAPT smears ranging from ∼50 to 250 kDa, indicative of NFT pathology. This pattern was absent in CN, FTD and PD tissues, supporting the specificity of tau aggregation to AD. Notably, a subset of AD samples (e.g., Sample ID 5955 in Figure 2A) lacked detectable high molecular weight MAPT signals, highlighting variability in NFT burden among AD cases. Interestingly, one CN individual displayed a characteristic MAPT smear in addition to the distinct 45–65 kDa bands, suggesting possible age-related tau accumulation or early signs of tauopathy (Figure 3A, red dotted rectangle).

### 3.3 MAPT levels are significantly elevated in AD hippocampus compared to age-matched CN, FTD, and PD individuals

Quantitative analysis of MAPT protein levels via immunoblotting revealed a significant increase in hippocampal MAPT abundance in both EOAD and LOAD cases compared to CN, FTD, and PD individuals (Figure 3B). To assess potential sex-based differences, MAPT levels were compared between male (n = 15/24/15) and female (n = 14/19/16) samples within the CN, EOAD, and LOAD groups, respectively. Unpaired t-tests showed no significant differences in MAPT abundance between sexes (CN: *p* = 0.2505; EOAD: *p* = 0.6829; LOAD: *p* = 0.4986; Supplementary Figure S1A), indicating that MAPT elevation in AD is not influenced by gender. Further, to evaluate whether MAPT levels are associated with age, protein abundance was plotted against age at death for both CN and AD individuals. Simple linear regression analysis demonstrated no significant correlation between age and MAPT levels in either group (CN: slope = −0.001924, *p* = 0.8908, R^2^ = 0.00071; AD: slope = −0.05673, *p* = 0.6058, R^2^ = 0.00377; Figures 3C,D). Regression analysis revealed no significant association between MAPT abundance and PMI. These findings suggest that the observed increase in MAPT protein in AD hippocampus is independent of age and sex.

### 3.4 CAMKK2 levels are significantly reduced in the hippocampus of AD, FTD, and PD patients compared to age-matched cognitively normal individuals

**FIGURE 4:**
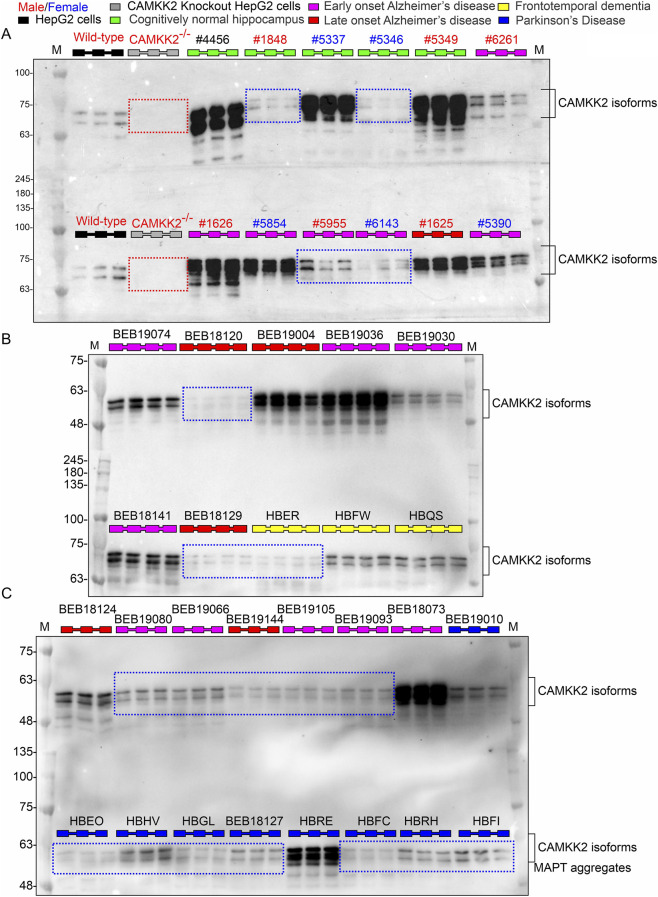
Abundance of CAMKK2 in hippocampal tissues from CN, EOAD, LOAD, FTD, and PD individuals (postmortem). **(A–C)** Representative immunoblots showing CAMKK2 protein levels in postmortem hippocampal tissues from CN, EOAD, LOAD, FTD, and PD individuals. Wild-type HepG2 cells and CAMKK2-knockout HepG2 cells were used as positive and negative controls, respectively. Red dotted rectangles indicate the absence of CAMKK2 protein in CAMKK2-knockout HepG2 cells. Blue dotted rectangles highlight low expression of CAMKK2 isoforms in select hippocampal samples from both non-demented and neurodegenerative cases. Notably, two CAMKK2 isoforms are consistently expressed across all tissue samples, previously identified as exon 14 alternatively spliced isoforms of CAMKK2 (Sabbir et al., 2020). Each sample was loaded in triplicates or quadruplicates. The corresponding MAPT expression profiles for these samples were previously shown in Figure 2.

To assess CAMKK2 protein levels across neurodegenerative conditions, hippocampal tissues from CN, EOAD, LOAD, FTD, and PD individuals were analyzed. Protein levels were normalized to CAMKK2 expression in wild-type HepG2 cells, which served as an internal reference across immunoblots to minimize inter-blot variability (Figure 4A). The use of HepG2 cells was based on prior studies involving CRISPR/Cas9-mediated CAMKK2 knockout lines, which were developed to investigate CAMKK2’s role in TF trafficking (Sabbir, 2018). Wild-type and CAMKK2-deleted HepG2 lysates were included as positive and negative controls, respectively (Figure 4A). Notably, this normalization strategy was not applied to MAPT quantification, as HepG2 cells do not express MAPT (Figure 2Ab blue dotted rectangles). Immunoblotting revealed a wide range of CAMKK2 expression across samples. While some CN hippocampus exhibited low CAMKK2 levels, reduced expression was also observed in subsets of AD, PD, and FTD samples (Figures 4A–C, blue dotted rectangles). CAMKK2 knockout HepG2 lysates confirmed antibody specificity and served as a negative control (Figure 4A, red dotted rectangles).

**FIGURE 5:**
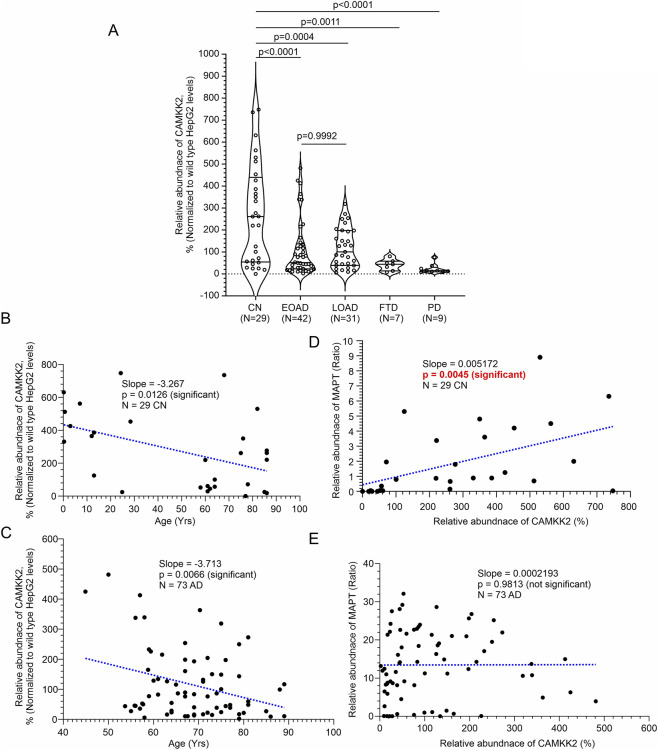
Relative abundance of CAMKK2 in hippocampal tissues from CN, EOAD, LOAD, FTD, and PD individuals, and correlation of CAMKK2 expression with MAPT levels and age in CN and AD patients (postmortem). **(A)** Scatter plot showing CAMKK2 protein levels normalized to Wild-type HepG2 cells in hippocampal tissues from 29 CN individuals, 42 EOAD, 31 LOAD, 7 FTD, and 9 PD patients (postmortem). Relative protein abundance (%) was calculated by comparing each sample to a reference wild-type HepG2 sample (set at 100%). Values represent the mean from three independent immunoblotting experiments, each with 3–4 replicates using the same tissue lysates. Data are presented as Mean ± SEM. Statistical significance was determined using Ordinary One-Way ANOVA followed by Dunnett’s Multiple Comparisons test. **(B,C)** Scatter plots showing CAMKK2 abundance plotted against age in CN individuals **(B)** and AD patients **(C)**. **(D,E)** Scatter plots showing CAMKK2 abundance plotted against MAPT levels in CN individuals **(D)** and AD patients **(E)**. Regression lines were generated using GraphPad Prism software.

Quantitative analysis revealed a significant reduction in CAMKK2 abundance in EOAD, LOAD, FTD, and PD hippocampal tissues compared to the CN group (Figure 5A), suggesting that CAMKK2 downregulation may be a common feature across a broad spectrum of neurodegenerative conditions. To assess potential sex-based differences, CAMKK2 levels were compared between male (n = 15/24/15) and female (n = 14/19/16) individuals within the CN, EOAD, and LOAD groups, respectively. Unpaired t-tests revealed no significant differences between sexes (CN: *p* = 0.7382; EOAD: *p* = 0.7227; LOAD: *p* = 0.7847; Supplementary Figure S1B), indicating that CAMKK2 reduction in AD is not sex-dependent. Similar analyses were not performed for PD and FTD groups due to limited sample sizes. To evaluate age-related effects, CAMKK2 levels were plotted against age at death for CN and AD individuals. Simple linear regression revealed a significant negative correlation between age and CAMKK2 expression in both groups (CN: slope = −3.267, *p* = 0.0126, R^2^ = 0.2092; AD: slope = −3.713, *p* = 0.0066, R^2^ = 0.0992; Figures 3C,D). However, multiple linear regression analysis showed that the rate of decline with age was statistically similar between groups, as indicated by the non-significant interaction term (*p* = 0.8188). Additionally, the group effect (AD versus CN) itself was not significant (*p* = 0.6242), while age remained a strong predictor of CAMKK2 expression (*p* = 0.0002). Regression analysis revealed no significant association between CAMKK2 abundance and PMI.

To further investigate the functional relationship between CAMKK2 and MAPT protein expression, correlation analyses were performed in CN and AD hippocampal samples. In the CN group, CAMKK2 and MAPT levels exhibited a moderate and statistically significant positive correlation (Pearson r = 0.5120, p = 0.0045), suggesting a potential regulatory relationship under normal physiological conditions (Figure 5D). In contrast, this association was absent in the AD group (Pearson r = 0.0027, p = 0.9813), indicating a disruption in potential CAMKK2-MAPT coupling in the context of Alzheimer’s pathology (Figure 5E). These findings imply that CAMKK2 may contribute to MAPT regulation in non-demented brains, and its dysregulation in AD could lead to MAPT-related abnormalities, such as hyperphosphorylation and aggregation.

Collectively, these findings suggest that while CAMKK2 expression in the hippocampus naturally declines with age, the overall reduction observed in AD is more pronounced and likely reflects neurodegenerative disease-specific pathophysiological mechanisms rather than age or sex alone. Furthermore, the loss of correlation between CAMKK2 and Tau expression in AD supports the hypothesis that CAMKK2 dysfunction may contribute to Tau dysregulation and neurodegeneration. This decoupling of CAMKK2-Tau interaction may represent a novel mechanistic link in AD pathology and warrants further investigation into CAMKK2 as a potential therapeutic target.

### 3.5 TF levels are significantly reduced in the hippocampus of AD, FTD, and PD patients compared to age-matched CN individuals

**FIGURE 6:**
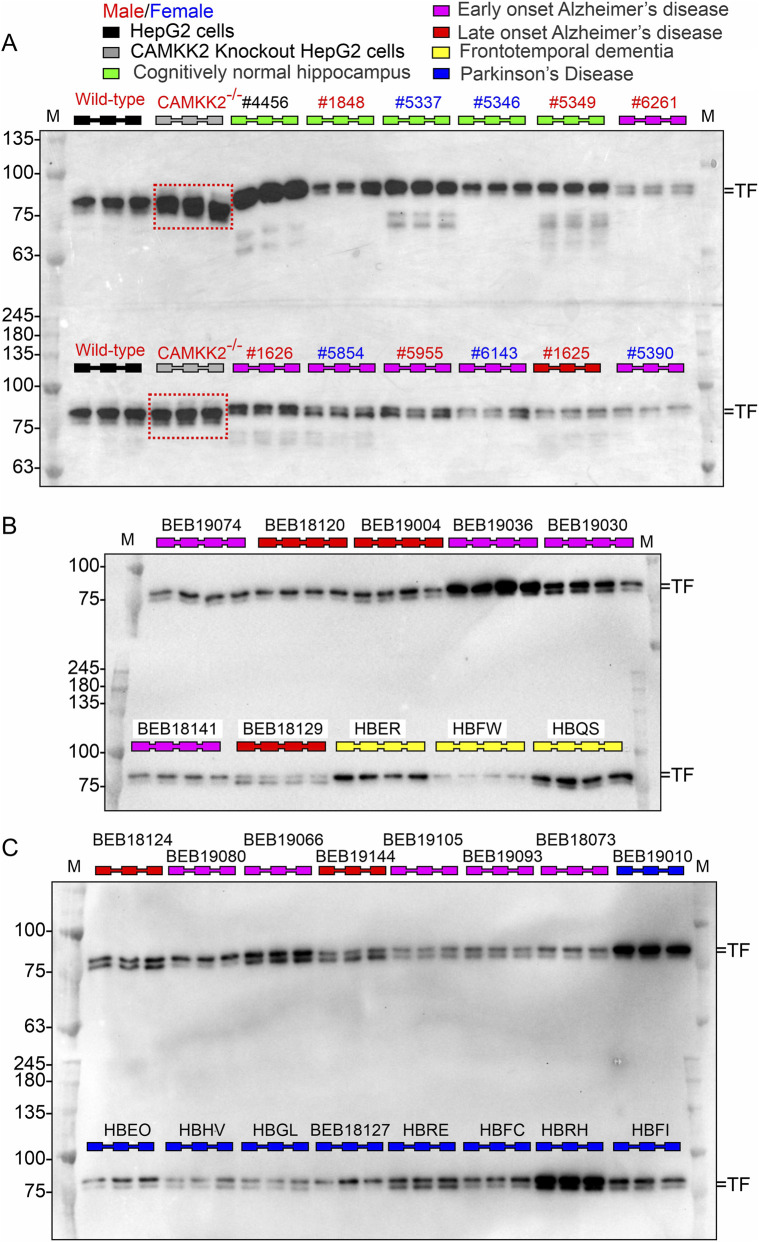
Abundance of TF in hippocampal tissues from CN, EOAD, LOAD, FTD, and PD individuals (postmortem). **(A–C)** Representative immunoblots showing TF protein levels in postmortem hippocampal tissues from CN, EOAD, LOAD, FTD, and PD individuals. Wild-type HepG2 cells and CAMKK2-knockout HepG2 cells were used as positive controls. Red dotted rectangles indicate a significant increase in TF content in CAMKK2-knockout HepG2 cells (151%) compared to wild-type HepG2 cells (set at 100%), consistent with previously reported findings using the same cell lines maintained over multiple passages across 6 years (Sabbir, 2018). Each sample was loaded in triplicates or quadruplicates. The corresponding MAPT and CAMKK2 expression profiles for these samples are shown in Figures 2, 4, respectively.

**FIGURE 7:**
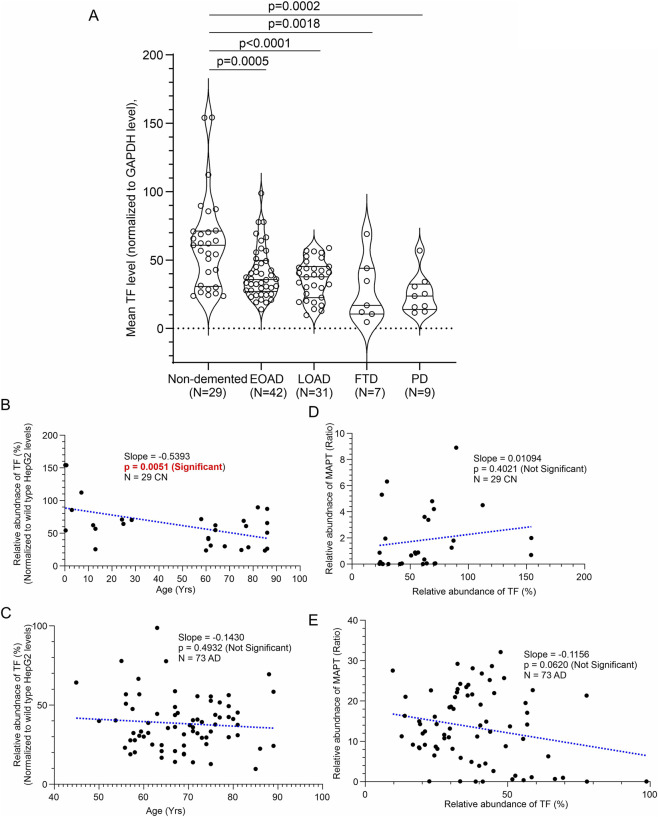
Relative abundance of TF in hippocampal tissues from CN, EOAD, LOAD, FTD, and PD individuals, and correlation of TF expression with MAPT levels and age in CN and AD patients (postmortem). **(A)** Scatter plot showing TF protein levels normalized to wild-type HepG2 cells in hippocampal tissues from 29 CN individuals, 42 EOAD, 31 LOAD, 7 FTD, and 9 PD patients (postmortem). Relative protein abundance (%) was calculated by comparing each sample to the reference wild-type HepG2 sample (set at 100%). Values represent the mean from three independent immunoblotting experiments, each with 3–4 replicates using the same tissue lysates. Data are presented as Mean ± SEM. Statistical significance was determined using Ordinary One-Way ANOVA followed by Dunnett’s Multiple Comparisons test. **(B,C)** Scatter plots showing TF abundance plotted against age in CN individuals **(B)** and AD patients **(C)**. **(D,E)** Scatter plots showing TF abundance plotted against MAPT levels in CN individuals **(D)** and AD patients **(E)**. Regression lines were generated using GraphPad Prism software.

TF protein levels in the CN, AD, FTD, and PD samples were analyzed by immunoblotting (Figures 6A–C). Wild-type and CAMKK2 knockout HepG2 cells were used as internal reference standards to normalize TF abundance across immunoblots (Figure 6A). This approach ensured consistency across experiments and was based on prior findings showing that TF levels are significantly elevated in CAMKK2-deficient HepG2 cells. This increase was consistently observed across multiple passages (Figure 6A, red dotted rectangles). TF levels in human hippocampal samples were normalized to wild-type HepG2 TF expression to account for inter-blot variability. Quantitative analysis revealed a significant reduction in TF abundance in EOAD, LOAD, FTD, and PD hippocampal tissues compared to CN samples (Figure 7A), suggesting that TF downregulation may be a shared feature across multiple neurodegenerative conditions.

To assess sex-based differences, TF levels were compared between male (n = 15/24/15) and female (n = 14/19/16) individuals within the CN, EOAD, and LOAD groups, respectively. Unpaired t-tests showed no significant differences between sexes (CN: *p* = 0.3361; EOAD: *p* = 0.4745; LOAD: *p* = 0.8887; Supplementary Figure S1C), indicating that TF reduction in AD is not sex-dependent. Similar analyses were not performed for PD and FTD groups due to limited sample sizes. To evaluate age-related effects, TF levels were plotted against age at death for CN and AD individuals. Simple linear regression revealed a significant negative correlation between age and TF expression in the CN group (*slope* = −0.5393, *p* = 0.0051, R^2^ = 0.2556), but no significant relationship in the AD group (*slope* = −0.1430, *p* = 0.4932, R^2^ = 0.0066; Figures 7B,C). A multiple linear regression model assessing the combined effects of age and group (CN vs. AD) showed that both age (*p* < 0.0001) and group (*p* = 0.005) were significant predictors of TF expression. However, the interaction term (*Age × Group*) was not significant (*p* = 0.1759), indicating that the rate of TF decline with age does not differ significantly between CN and AD groups. Regression analysis revealed no significant association between TF abundance and PMI.

To further explore the functional relationship between TF and MAPT protein expression, Pearson correlation analyses were performed in CN and AD hippocampal samples. In the CN group, TF and MAPT levels showed a weak positive correlation that was not statistically significant (*r* = +0.1617, *p* = 0.4021), suggesting no clear association. In contrast, the AD group exhibited a trend toward a negative correlation (*r* = −0.2196, *p* = 0.06196), indicating that higher TF expression may be associated with lower MAPT levels, although this trend did not reach statistical significance.

Additionally, Pearson correlation analyses were performed in CN and AD hippocampal samples to explore the functional relationship between TF and CAMKK2 protein expression, In the CN group, TF and CAMKK2 levels showed statistically significant positive correlation that was (*r = 0.541, p = 0.0025*), in AD group such correlation was weak, and non-significant (r = 0.173, p = 0.144). TF and CAMKK2 are positively associated overall and in controls; the relation is much weaker (and not significant) in the disease group. Disease is associated with lower TF and lower CAMKK2 overall. These findings indicate that Higher CAMKK2 expression aligns with higher TF levels overall, but in disease state this coupling appears attenuated.

### 3.6 TFRC levels are significantly reduced in the LOAD hippocampus, but not in LOAD, FTD, and PD patients compared to age-matched CN individuals

**FIGURE 8:**
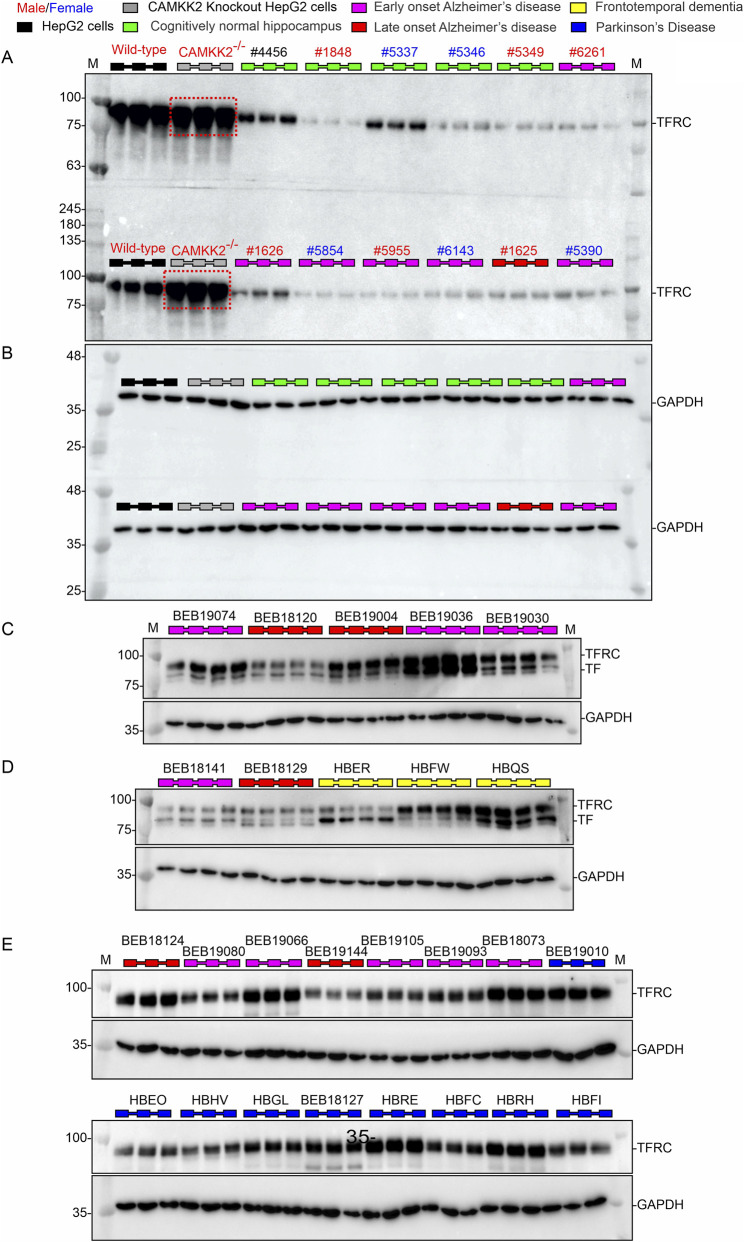
Abundance of TFRC in hippocampal tissues from CN, EOAD, LOAD, FTD, and PD individuals (postmortem). **(A–E)** Representative immunoblots showing TFRC and GAPDH protein levels in postmortem hippocampal tissues from CN, EOAD, LOAD, FTD, and PD individuals. Wild-type HepG2 cells and CAMKK2-knockout HepG2 cells were used as positive controls. Red dotted rectangles indicate a significant increase in TF content in CAMKK2-knockout HepG2 cells (115%) compared to wild-type HepG2 cells (set at 100%), consistent with previously reported findings using the same cell lines maintained over multiple passages across 6 years (Sabbir, 2018). Each sample was loaded in triplicates or quadruplicates. The corresponding MAPT, CAMKK2, and TF expression profiles for these samples are shown in Figures 2, 4, 6, respectively.

**FIGURE 9:**
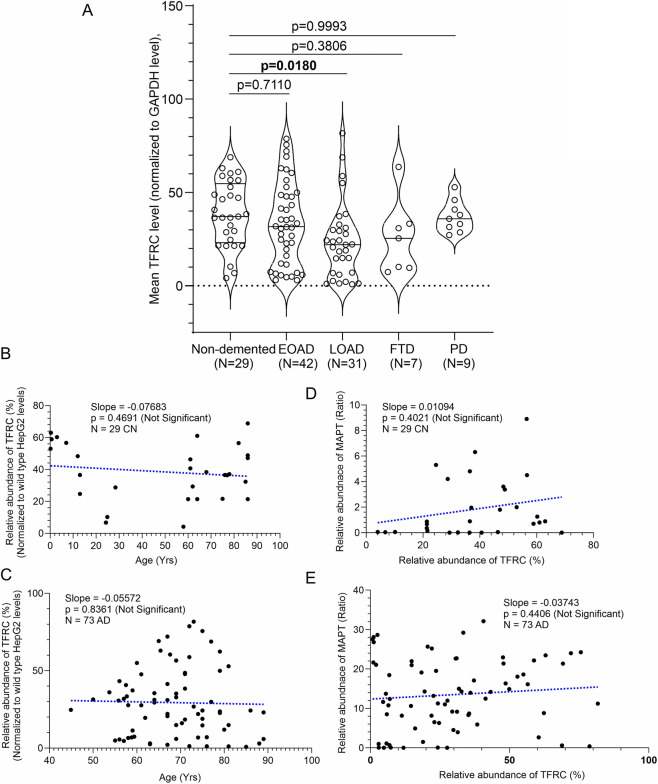
Relative abundance of TFRC in hippocampal tissues from CN, EOAD, LOAD, FTD, and PD individuals, and correlation of TF expression with MAPT levels and age in CN and AD patients (postmortem). **(A)** Scatter plot showing TFRC protein levels normalized to wild-type HepG2 cells in hippocampal tissues from 29 CN individuals, 42 EOAD, 31 LOAD, 7 FTD, and 9 PD patients (postmortem). Relative protein abundance (%) was calculated by comparing each sample to the reference wild-type HepG2 sample (set at 100%). Values represent the mean from three independent immunoblotting experiments, each with 3–4 replicates using the same tissue lysates. Data are presented as Mean ± SEM. Statistical significance was determined using Ordinary One-Way ANOVA followed by Dunnett’s Multiple Comparisons test. **(B,C)** Scatter plots showing TFRC abundance plotted against age in CN individuals **(B)** and AD patients **(C)**. **(D,E)** Scatter plots showing TF abundance plotted against MAPT levels in CN individuals **(D)** and AD patients **(E)**. Regression lines were generated using GraphPad Prism software.

TFRC protein levels were assessed in hippocampal samples from CN, AD, FTD, and PD cases by immunoblotting (Figures 8A–C). Wild-type and CAMKK2 knockout HepG2 cells served as internal reference standards for normalization across blots (Figure 8A). Notably, CAMKK2 deletion significantly increased TFRC abundance in HepG2 cells, suggesting that CAMKK2 may regulate receptor-mediated transferrin trafficking in liver-derived cells (Figure 8A, red dotted rectangles). Quantitative analysis revealed a significant reduction in TFRC abundance in late-onset AD (LOAD) hippocampal tissue (*p* = 0.0180), whereas no significant differences were observed in early-onset AD (EOAD), FTD, or PD compared with CN (*p* = 0.7110, 0.3806, and 0.9993, respectively; Figure 9A). These findings suggest that TFRC downregulation may represent a late event in AD progression rather than a shared feature across multiple neurodegenerative disorders.

Sex-based comparisons indicated no significant differences in TFRC levels between males and females within CN, EOAD, or LOAD groups (CN: *p* > 0.9999; EOAD: *p* = 0.7785; LOAD: *p* = 0.8488; Supplementary Figure S1D). Analyses for PD and FTD were not performed due to limited sample sizes. Examination of age–TFRC associations in CN and AD samples showed no significant relationships (CN: *r* = −0.14, *p* = 0.469; AD: *r* = −0.025, *p* = 0.836), indicating that TFRC expression is not age-dependent in these cohorts. The relationships between MAPT, CAMKK2, and TFRC were also evaluated in CN and AD groups. Within-group correlations between MAPT and TFRC were small and not statistically significant (CN: Pearson *r* = 0.236, *p* = 0.218; AD: Pearson *r* = 0.092, *p* = 0.441). Similarly, CAMKK2–TFRC correlations were not significant (CN: Pearson *r* = 0.178, *p* = 0.355; AD: Pearson *r* = −0.229, *p* = 0.052). Regression analysis revealed no significant association between MAPT abundance and PMI. Collectively, these findings indicate no statistically reliable association between MAPT or CAMKK2 and TFRC within either group and no evidence of a disease-specific interaction among these variables.

### 3.7 IEF revealed no significant alterations in CAMKK2 and TF charge distribution in EOAD hippocampus

Our previous studies using CAMKK2 knockout models - including transformed human cancer cell lines and Camkk2-deficient mice - demonstrated a selective loss of a negatively charged fraction of TF, which was associated with altered trafficking and impaired iron homeostasis (Sabbir, 2020; Sabbir, 2018). To investigate whether similar changes occur in the human hippocampus during AD pathogenesis, we performed isoelectric focusing IEF on postmortem tissue samples from two CN individuals and two EOAD patients. The pH gradient ranged from 3 to 10, allowing resolution of protein species based on their isoelectric points. CAMKK2 was detected across multiple fractions in all samples, with a consistent presence in the more positively charged regions. No discernible shift or loss of specific isoforms was observed between CN and EOAD samples. Similarly, TF appeared predominantly in a single negatively charged fraction across all individuals, with no significant variation in its distribution pattern between CN and EOAD groups. These findings suggest that, despite the overall reduction in CAMKK2 and TF protein abundance reported in EOAD, their charged profiles remain largely unaltered in the hippocampus.

## 4 Discussion

This retrospective human postmortem tissue-based study provides descriptive evidence that CAMKK2 and its associated iron transport proteins - TF and TFRC - are significantly reduced in the hippocampus of individuals with AD compared to CN controls. These reductions were observed alongside elevated MAPT (tau) protein abundance and aggregation, a hallmark of AD pathology. Importantly, the observed associations are correlative and do not establish direct mechanistic links among CAMKK2, TF, TFRC, and tau pathology. Correlation analyses revealed that CAMKK2 and MAPT levels were positively associated in CN hippocampus but not in AD, suggesting a disease-specific disruption of their normal relationship. Similarly, CAMKK2 and TF showed a strong positive correlation in CN samples, which was attenuated in AD. These findings indicate that CAMKK2 downregulation coincides with alterations in iron transport proteins and tau aggregation in AD, but the nature of these relationships remains unclear.

Our previous work using CRISPR/Cas9-mediated CAMKK2 knockout cell lines (Sabbir et al., 2020; Sabbir, 2018) and Camkk2 knockout mouse models (Sabbir, 2020) provided mechanistic evidence that CAMKK2 regulates transferrin trafficking and iron homeostasis through its downstream kinase effector, CAMK4 signaling. These experimental systems allowed controlled manipulation of CAMKK2, enabling us to establish causal relationships between calcium signaling and iron transport. The current postmortem findings complement these models by confirming that CAMKK2, TF, and TFRC are significantly reduced in the cortex (Sabbir, 2024) and hippocampus of AD patients, thereby validating that the molecular alterations observed *in vitro* and *in vivo* are disease-relevant and not experimental artifacts. However, the underlying mechanisms and cell-type specificity of this downregulation remain unclear and warrant further investigation. Given that CAMKK2 is expressed in various brain cell types - including neurons, astrocytes, and microglia (Sabbir, 2024) - future studies should aim to dissect the contribution of each cell population to the observed changes. A promising approach would be large-scale immunohistochemical analysis of fixed postmortem human hippocampus tissues to identify the specific cell types exhibiting CAMKK2 loss. Neurons are likely key contributors, as early synaptic dysfunction—particularly the loss of excitatory synapses in the hippocampus and cortex - is a well-documented hallmark of AD (Davies et al., 1987; Masliah et al., 2001; Moolman et al., 2004). Moreover, soluble Aβ42 dimers and oligomers isolated from AD brains have been shown to directly induce Tau hyperphosphorylation and neuritic degeneration (Jin et al., 2011). The CAMKK2–AMPK signaling pathway has been implicated in mediating Aβ oligomer-induced synaptic loss (Mairet-Coello et al., 2013; Lee et al., 2022), further supporting a neuronal role in CAMKK2-related pathology. Nonetheless, the involvement of glial cells—especially astrocytes and microglia—should not be overlooked (Di Benedetto et al., 2022). Astrocytes play critical roles in maintaining BBB integrity, regulating cerebral blood flow, providing metabolic and trophic support to neurons, buffering ions, clearing neurotransmitters, and modulating synaptic transmission and plasticity (Abbott et al., 2006; Vesce et al., 1999). These functions are tightly coupled to astrocytic Ca^2+^ signaling, which is disrupted in AD. For instance, local application of Aβ oligomers has been shown to elevate astroglial Ca^2+^ levels in acute brain slices (Tyurikova et al., 2018) and amyloid pathology alters astrocyte Ca^2+^ dynamics in the 5XFAD mouse model of familial AD (Weiss et al., 2023). Microglia also exhibit altered Ca^2+^ homeostasis in AD. Using Ca^2+^-sensitive fluorescence microscopy, McLarnon et al. demonstrated abnormal Ca^2+^ signaling in microglia from AD brains compared to CN individuals, findings that were replicated in human fetal microglia exposed to pathogenic Aβ42 peptides (McLarnon et al., 2005). In summary, future studies employing cell-type-specific analyses - particularly large-scale immunohistochemistry and single-cell transcriptomics - are essential to determine which hippocampal and cortical cell populations are most susceptible to altered calcium/iron homeostasis and CAMKK2 downregulation in AD.

Importantly, the observed reduction in CAMKK2 protein levels was not exclusive to AD but was also evident in hippocampal tissues from individuals with FTD and PD, suggesting that CAMKK2 downregulation may represent a shared molecular feature across multiple neurodegenerative disorders. The inclusion of FTD (n = 7) and PD (n = 9) cases was intended to provide comparative context; however, these sample sizes are limited and were not powered for robust statistical inference. Findings from this cohort of samples should be interpreted cautiously, and future studies will require larger cohorts or multi-center collaborations to validate these observations. PD, the second most prevalent neurodegenerative disease, is characterized by pathological aggregation of α-synuclein (Zaichick et al., 2017), while FTD is most commonly associated with TAR DNA-binding protein 43 (TDP-43) proteinopathies (Cairns et al., 2007; Grossman et al., 2023). Over the past decade, extensive genetic studies have identified more than 30 loci associated with PD, many of which are linked to Ca^2+^ homeostasis (Zaichick et al., 2017). A key pathological consequence of α-synuclein aggregation is the disruption of intracellular Ca^2+^ signaling (Zaichick et al., 2017; Caraveo et al., 2014). Similarly, elevated intracellular Ca^2+^ levels have been implicated in TDP-43-mediated neurotoxicity, as demonstrated in a *Caenorhabditis elegans* model expressing mutant TDP-43 (A315T) in motor neurons (Aggad et al., 2014). Moreover, activation of voltage-gated calcium channels (VGCCs) promotes Ca^2+^ influx, and in one case study, autoantibodies targeting P/Q- and N-type VGCCs have been reported to mimic FTD-like symptoms (Younes et al., 2018). Collectively, these findings underscore a mechanistic link between dysregulated calcium signaling and the pathogenesis of PD and FTD (Ureshino et al., 2019). Which may be mechanistically tied to the observed CAMKK2 downregulation in these conditions. Further research is needed to elucidate whether CAMKK2 serves as a convergent node in Ca^2+^/CAM-dependent neurodegenerative pathways (Bohush et al., 2021) across distinct disease entities.

An important question arising from the findings of these studies (Sabbir, 2024) is whether the observed reduction in CAMKK2 protein levels in hippocampal tissues is driven by altered transcriptional regulation or post-translational mechanisms. Although transcriptional downregulation of CAMKK2 has been previously reported, it was observed in monocytic cells rather than brain cells. Specifically, our group demonstrated that phorbol ester-induced differentiation of human monocytic THP-1 cells into macrophage-like cells led to significant transcriptional downregulation of CAMKK2, resulting in a marked reduction of CAMKK2 protein compared to vehicle-treated cells (Sabbir et al., 2020). This raises the possibility that Aβ-induced activation of brain immune cells in AD could exert a similar effect. However, this hypothesis may be challenged by transcriptomic data from human brain. A *post hoc* analysis of the publicly available GEO dataset (accession number: GSE44770), which includes a large-scale cross-study transcriptomic comparison of late-onset AD brains (129 samples) and age-matched non-demented controls (101 samples), revealed no significant reduction in CAMKK2 mRNA expression in the prefrontal cortex of AD patients. Interestingly, CAMK4 expression was significantly decreased, suggesting that while CAMKK2 transcription may remain stable, dysfunction in the CAMKK2–CAMK4 signaling axis could contribute to AD pathology. Previously we demonstrated that this axis regulates calcium and iron homeostasis (Sabbir, 2020), both of which are intricately linked to neurodegeneration in AD. Therefore, future studies should employ cell-type-specific analyses of CAMKK2 gene expression using fresh or rapidly preserved human tissues to determine whether its loss reflects transcriptional downregulation. Meta-analysis of existing single-nucleus RNA sequencing (snRNA-seq) datasets available through platforms such as CELLxGENE (Abdulla et al., 2025) or UCSC Cell Browser (Speir et al., 2021), as well as bulk RNA expression datasets in the Gene Expression Omnibus (GEO) (Clough and Barrett, 2016) could provide initial insights into transcriptional changes. At the post-transcriptional level, miRNA-mediated regulation may play a role. For instance, miR-378a-5p has been shown to bind to the 3′UTR of CAMKK2 and downregulate its expression in rat hippocampal neurons, although its relevance to AD remains unclear (Zhang et al., 2021). Additionally, genetic variation may influence CAMKK2 expression: the T variant of the SNP rs1063843 has been associated with CAMKK2 downregulation in individuals with schizophrenia [Luo, 2014], though the underlying mechanism is not yet understood (Luo et al., 2014).

Given the lack of transcriptional changes, post-translational mechanisms may play a more prominent role in CAMKK2 downregulation. One possibility is that altered post-translational modifications - such as phosphorylation - may trigger ubiquitination and subsequent proteasomal degradation of CAMKK2. Although current study did not detect significant differences in CAMKK2 charge states, this may be due to the rapid dephosphorylation of proteins during postmortem intervals. Indeed, most proteins in mouse brain, heart, liver, and kidney have been shown to undergo varying degrees of dephosphorylation within 20 s to 10 min postmortem (Wang et al., 2015), making postmortem tissues less suitable for studying phosphorylation-dependent modifications. Nonetheless, CAMKK2 is known to be regulated by its phosphorylation state (Tokumitsu and Sakagami, 2022), and phosphorylation can serve as a signal for ubiquitination and proteasomal degradation. Supporting this, previous studies have shown that the stability of CaM-activated kinase 1 (CAMK1) is controlled by the E3 ligase Fbxl12, which facilitates its degradation via ubiquitination (Mallampalli et al., 2013). Interestingly, CAMKK2 has been reported to interact with ubiquitin ligases and 26S proteasome regulatory subunits (Ding and Sankar, 2024), and high-throughput mass spectrometry data archived in PhosphoSitePlus indicate multiple ubiquitination sites on CAMKK2 (Sabbir, 2020). This raises the possibility that CAMKK2 may be regulated by the ubiquitin–proteasome system through post-translational mechanisms. Future work should explore this hypothesis using mass spectrometry-based proteomics on fresh or minimally delayed postmortem tissues to detect ubiquitination and phosphorylation patterns. Additionally, proteasome activity assays and ubiquitination profiling in AD-relevant models could clarify whether CAMKK2 degradation is proteasome-mediated. Complementary studies using rodent AD models, human iPSC-derived neurons, or brain organoids will be valuable for dissecting transcriptional versus post-translational regulation under controlled conditions. These approaches, combined with phosphoproteomic and transcriptomic analyses, will help elucidate the mechanisms underlying CAMKK2 loss and its contribution to AD pathogenesis.

TF levels were significantly reduced in AD, FTD, and PD hippocampi, consistent with prior cortical findings (Sabbir, 2024). Correlation analyses revealed a positive association between CAMKK2 and TF levels in CN hippocampus, an association that was notably attenuated in AD. This supports the hypothesis that CAMKK2 may regulate TF expression or trafficking under normal physiological conditions, and that its loss in AD disrupts this coupling, potentially contributing to iron dyshomeostasis. Although TFRC did not show significant correlations with CAMKK2 or MAPT, its selective reduction in LOAD - while remaining unchanged in EOAD, FTD, and PD - suggests a role in disease progression rather than initiation. Supporting this, studies using [^125^I]TF binding to TFRC in human and rat brains have demonstrated that TFRC densities are significantly reduced in the hippocampus, temporal, and occipital cortices of AD patients, while remaining unchanged in the frontal and parietal cortices and cerebellum (Kalaria et al., 1992), consistent with our current findings. The overall significance and potential mechanisms underlying the observed downregulation of CAMMK2-TFRC/TF pathway and its impact on iron dyshomeostasis in AD have been discussed in detail in our previous publication (Sabbir, 2024), which examined similar findings in cortical tissues from AD patients.

Although direct measurement of iron content in our hippocampal samples was not feasible due to limited availability, converging evidence from prior studies highlights the relevance of iron accumulation in AD pathology. Iron deposition and distribution across the hippocampus have been linked to episodic memory performance in older adults at risk for AD (Zhou et al., 2024). A recent study employing quantitative susceptibility mapping (QSM) MRI and PET imaging demonstrated that increased tissue magnetic susceptibility - a proxy for iron content - is a significant predictor of mild cognitive impairment onset and cognitive decline, particularly in individuals with amyloid pathology (Chen et al., 2025). Furthermore, iron-accumulating microglia have been identified as the predominant Aβ-plaque-infiltrating immune cells in individuals with high Aβ and tau burden, suggesting that microglial iron may influence their functional phenotype in the context of AD (Kenkhuis et al., 2021). In an MRI-based study involving AD patients and CN individuals, hippocampal tissue damage was found to co-occur with increased ferritin-bound iron accumulation, contrasting with iron-resistant regions such as the thalamus (Raven et al., 2013). Additional mechanistic insight comes from *in vivo* imaging using metal ion–specific fluorescent turn-on sensors capable of simultaneously monitoring Fe^2+^ and Fe^3+^ (Wu et al., 2023). Wu et al. demonstrated a decreased Fe^3+^/Fe^2+^ ratio during ferroptosis and an increased Fe^3+^/Fe^2+^ ratio in the brains of AD model mice (Wu et al., 2023). Notably, this elevated ratio was predominantly observed in amyloid plaque regions, suggesting a correlation between plaque pathology and the accumulation or oxidation of iron. Taken together, these findings support the hypothesis that dysregulation of the CAMKK2–TFRC/TF axis may contribute to iron dyshomeostasis in the AD hippocampus, potentially exacerbating neurodegenerative processes through altered iron metabolism and microglial reactivity.

In our study, no significant sex differences were observed in CAMKK2, TF, TFRC, or MAPT abundance. However, sex-based differences in AD are well-documented in epidemiological studies, with women showing higher prevalence and often faster cognitive decline (Rahman et al., 2019), potentially influenced by hormonal (Uddin et al., 2020) and calcium signaling (Chami, 2021) pathways. For example, estrogen has been shown to induce a rapid increase in the activity of CAMKK2-related proteins, such as calmodulin-dependent protein kinase II, in the hippocampus. Emerging evidence also suggests sex-specific differences in brain iron metabolism in AD; a recent postmortem study reported that iron deposits and microglial ferritin levels were positively correlated in men but negatively correlated in women, indicating distinct iron-handling mechanisms between sexes (Rahman et al., 2025). These findings highlight the need for future research that integrates longitudinal neuroimaging, plasma/CSF biomarker analyses, and hormone profiling to better elucidate sex-specific mechanisms underlying CAMKK2–iron signaling and AD progression. Postmortem tissue analysis cannot capture dynamic physiological processes such as estrogen fluctuations or immune responses, reinforcing the importance of these complementary approaches.

The loss of correlation between CAMKK2 and MAPT in AD - contrasted with their positive association in CN - points to a potential disease-specific disruption of CAMKK2-mediated tau homeostasis. While our analyses quantified total MAPT and its aggregation, pathogenic phosphorylated MAPT (p-Tau) species are tightly linked to neurofibrillary tangle formation and neuronal dysfunction; hyperphosphorylation drives tau detachment from microtubules, misfolding, aggregation, and synaptic loss (Gong and Iqbal, 2008). Consistent with this, the CAMKK2–CAMK4 axis has been implicated in tau-phosphorylation pathways, providing a plausible route by which CAMKK2 loss could favor tau hyperphosphorylation and aggregation (Mairet-Coello et al., 2013; Wei et al., 2018). In our cohort, the smeared MAPT pattern - indicative of aggregated species - was prominent in AD but absent in PD and FTD, underscoring the AD specificity of tauopathy. Mechanistically, increased MAPT in AD likely reflects post-translational regulation, including abnormal hyperphosphorylation (Gong and Iqbal, 2008), ubiquitination (Li et al., 2021), and impaired degradation via the ubiquitin–proteasome and autophagy–lysosome pathways (Wang et al., 2025). Given the significant reduction of CAMKK2 in AD hippocampus and the decoupling from total MAPT, future work should test whether CAMKK2 downregulation directly promotes pathological tau phosphorylation and aggregation. Elucidating this link would clarify how calcium signaling dysregulation intersects with tau pathology and, together with our prior evidence that CAMKK2–CAMK4 signaling modulates iron homeostasis (Sabbir, 2020), positions CAMKK2 as a convergent therapeutic target across tauopathy and iron dyshomeostasis in AD.

In summary, this study demonstrates that CAMKK2 and TF are consistently reduced in the hippocampus of individuals with AD, FTD, and PD, while TFRC reduction is specific to late-onset AD. These changes occur alongside increased tau protein abundance and aggregation in AD, highlighting a pattern of molecular alterations associated with neurodegenerative pathology. However, these findings are descriptive and correlative; they do not establish cause-and-effect relationships among CAMKK2, iron transport proteins, and tau pathology. Future mechanistic studies are needed to determine whether CAMKK2 plays a direct role in regulating iron homeostasis or tau aggregation and to explore its potential as a therapeutic target.

## Data Availability

The raw data supporting the conclusions of this article will be made available by the authors, if requested, without undue reservation.

## Supplementary material

SUPPLEMENTARY FIGURE S1 MAPT, CAMKK2, TF, and TFRC protein levels in hippocampal tissues from male and female CN, EOAD, LOAD, FTD, and PD individuals (postmortem). **(A–D)** Scatter plots showing the mean protein levels of MAPT **(A)**, CAMKK2 **(B)**, TF **(C)**, and TFRC **(D)** in hippocampal tissues from 29 cognitively normal (CN) individuals, 42 early-onset Alzheimer’s disease (EOAD), 31 late-onset Alzheimer’s disease (LOAD), 7 frontotemporal dementia (FTD), and 9 Parkinson’s disease (PD) patients, segregated by sex to assess the effect of gender on protein abundance. These datasets correspond to those presented in Figures 3B, 5A, 7A, 9A, respectively. Data are presented as Mean ± SEM. Statistical significance was determined using Ordinary One-Way ANOVA followed by Dunnett’s Multiple Comparisons test.

SUPPLEMENTARY FIGURE S2 Correlation between CAMKK2, TF, and TFRC protein levels in hippocampal tissues from CN and AD individuals (postmortem). **(A,B)** Scatter plots showing the correlation between CAMKK2 and TF protein abundance in CN individuals **(A)** and AD patients **(B)**. **(C,D)** Scatter plots showing the correlation between CAMKK2 and TFRC protein abundance in CN individuals **(C)** and AD patients **(D)**. Regression lines were generated using GraphPad Prism software.

SUPPLEMENTARY FIGURE S3 Isoelectric focusing of CAMKK2 and TF proteins in postmortem hippocampal tissues from CN individuals and EOAD patients. **(A–D)** Immunoblots showing charge-separated fractions of CAMKK2 protein in CN samples (#4456 and #5337; panels **(A,B)** and EOAD samples (#3939 and #4138; panels **(C,D)**. **(E–H)** Immunoblots showing charge-separated fractions of TF protein in the same CN (panels **(E,F)**) and EOAD (panels **(G,H)**) samples. Colored dotted rectangles indicate distinct charge-based fractions of the proteins across the pH gradient (pH 3–10), representing native as well as post-translationally modified forms. Note: The theoretical isoelectric points (pI) of the two major CAMKK2 isoforms are approximately 6.25 and 7.53, respectively (Sabbir et al., 2020), while the pI of TF is approximately 6.81 (Sabbir, 2018). In previous studies, we reported that loss of CAMKK2 significantly reduced a negatively charged fraction of TF, which was associated with altered transferrin trafficking and iron homeostasis (Sabbir, 2018).
