# Supplementary figures and images for: Loss of CAMKK2 and iron-transport proteins—transferrin and its receptor—in the Alzheimer’s disease hippocampus: link to tau pathology
Source: Front Cell Dev Biol. 2026 Jan 22;14:1716718. doi: 10.3389/fcell.2026.1716718 (PMC12872923; doi:10.3389/fcell.2026.1716718)

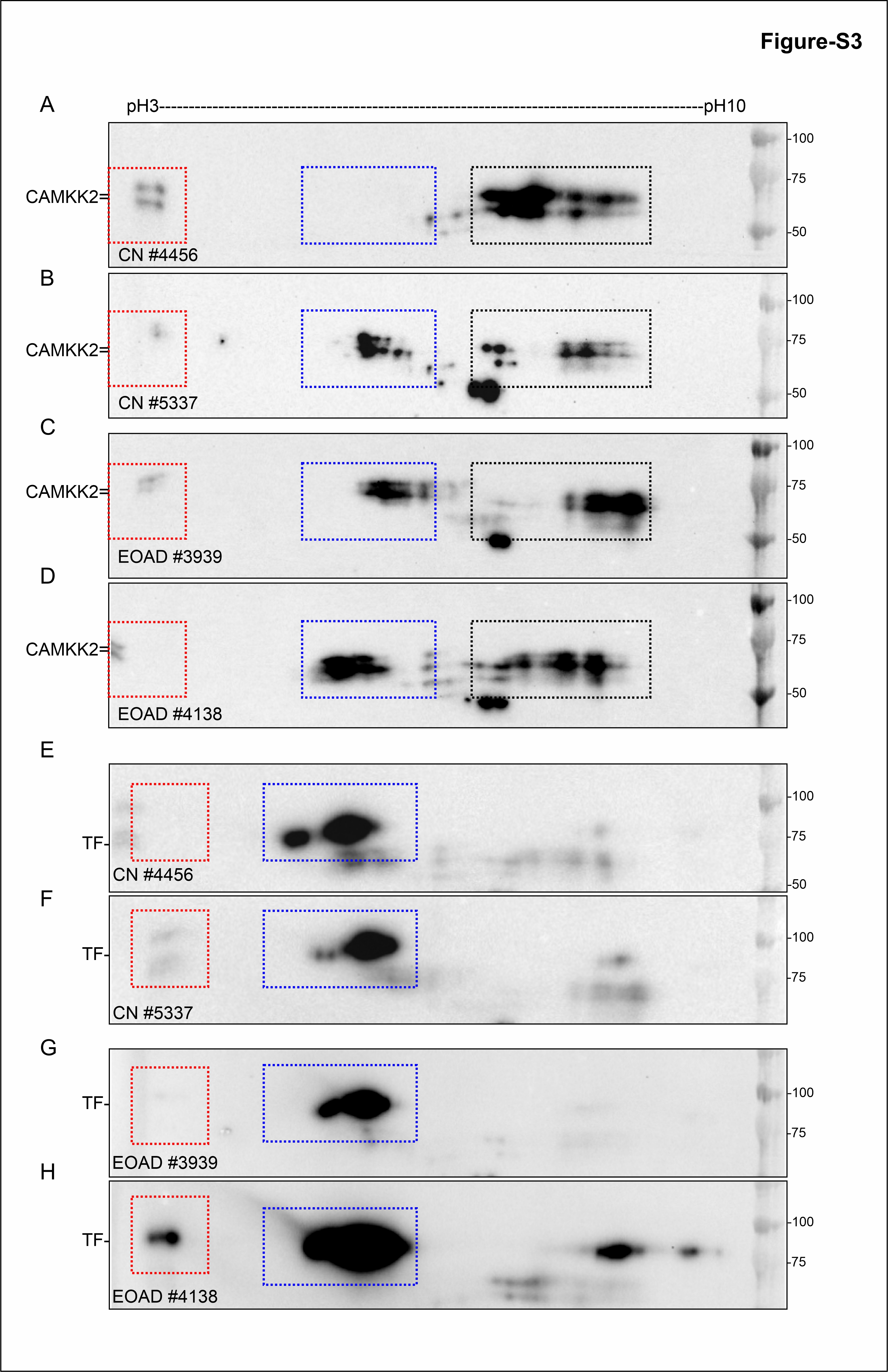

Supplement: Supplementary file 1 [file Image3.jpeg]

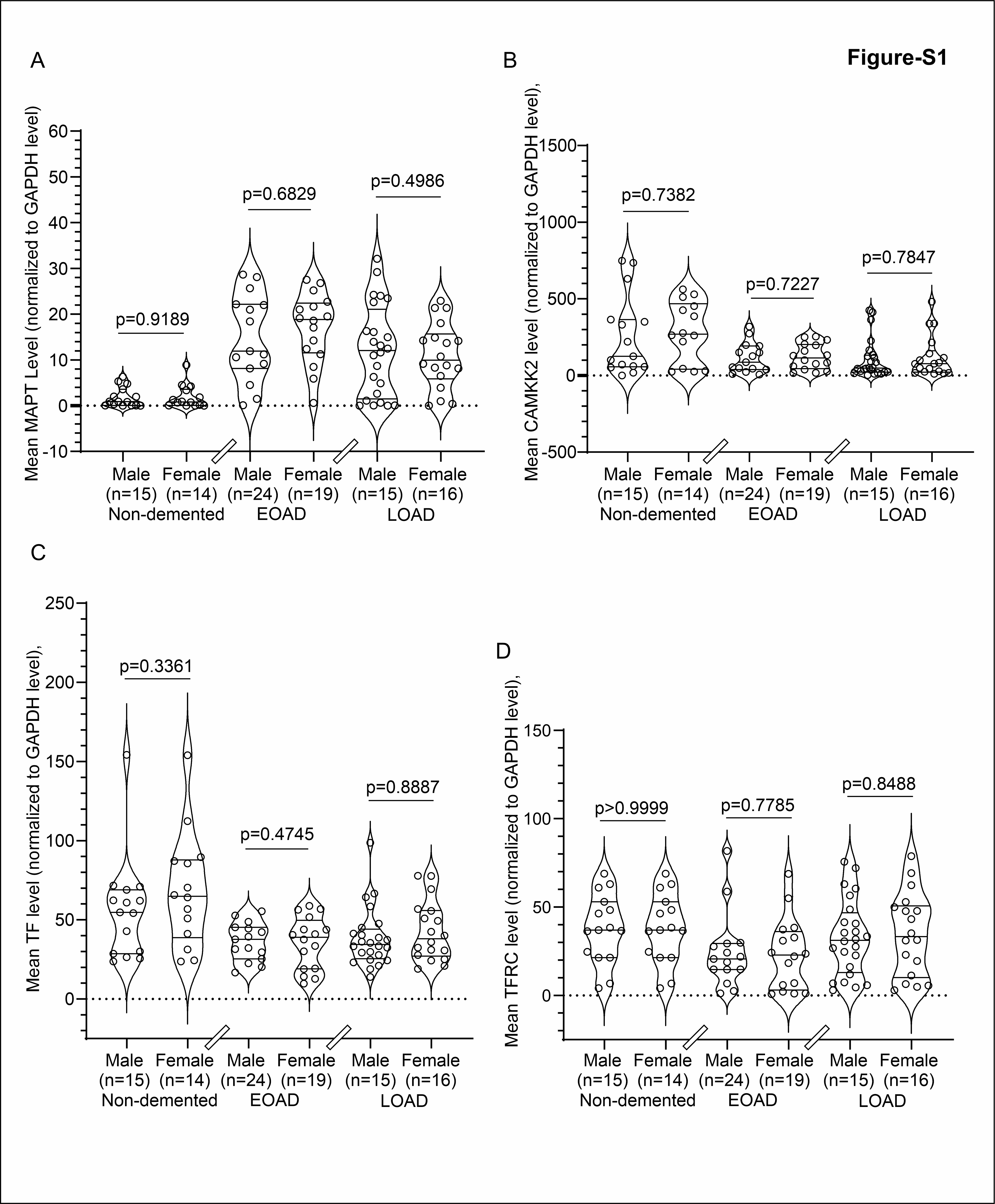

Supplement: Supplementary file 3 [file Image1.jpeg]

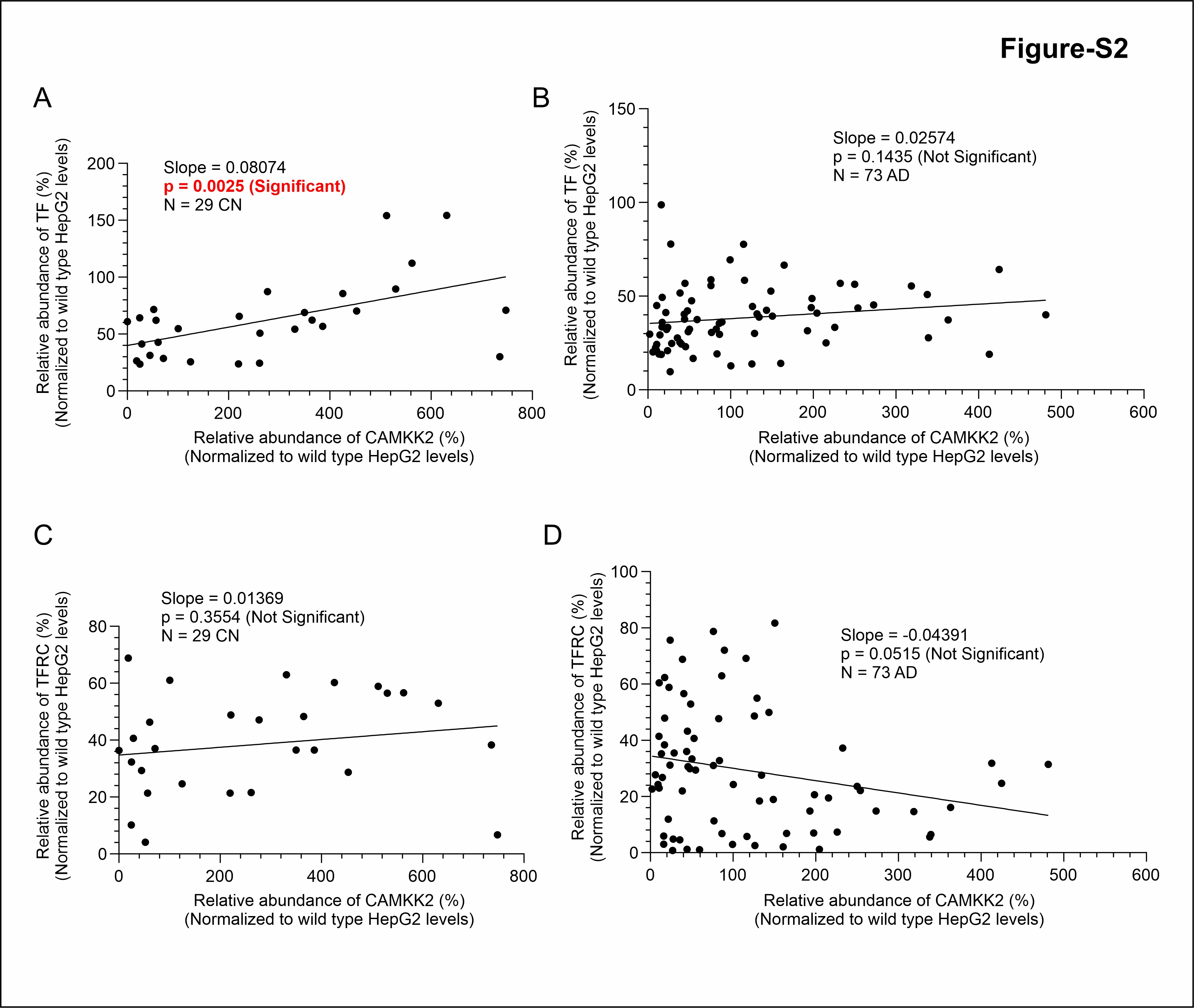

Supplement: Supplementary file 4 [file Image2.jpeg]
