# Supplementary material for: Loss of CAMKK2 and iron-transport proteins—transferrin and its receptor—in the Alzheimer’s disease hippocampus: link to tau pathology
Source: Front Cell Dev Biol. 2026 Jan 22;14:1716718. doi: 10.3389/fcell.2026.1716718 (PMC12872923; doi:10.3389/fcell.2026.1716718)
Supplement: Supplementary file 2 [file Table1.docx]

| Sl No | NIH NBB ID | Repository | Age at Death (Years) | Brain Region | Sex | PMI (Hours) |
| --- | --- | --- | --- | --- | --- | --- |
| 1 | 4711 | Maryland | 0.356 | Hippocampus | Male | 1 |
| 2 | 4644 | Maryland | 0.69 | Hippocampus | Female | 1 |
| 3 | 4441 | Maryland | 3 | Hippocampus | Female | 15. |
| 4 | **5925** | Maryland | 7 | Hippocampus | Female | 30 |
| 5 | 6278 | Maryland | 12 | Hippocampus | Male | 37 |
| 6 | HCTZZQ | Miami | 13 | Hippocampus | Female | 18.2 |
| 7 | 5376 | Maryland | 13 | Hippocampus | Male | 19 |
| 8 | 6054 | Maryland | 76 | Hippocampus | Male | 16.3 |
| 9 | 6072 | Maryland | 82 | Hippocampus | Female | 21 |

**Supplemetatry Table 1: Sample Information for Cognitively Normal Human Hippocampal Tissues Obtained from the NIH NeuroBioBank**
